# Supplementary material for: Prior infection with unrelated neurotropic virus exacerbates influenza disease and impairs lung T cell responses
Source: Nat Commun. 2024 Mar 23;15:2619. doi: 10.1038/s41467-024-46822-7 (PMC10960853; doi:10.1038/s41467-024-46822-7)
Supplement: Supplementary file 1 — Supplementary Information [file 41467_2024_46822_MOESM1_ESM.pdf]

SUPPLEMENTARY INFORMATION

Prior infection with an unrelated virus exacerbates influenza disease and impairs lung T cell responses

Isabelle Jia-Hui Foo[1,2], Brendon Y. Chua[1], E. Bridie Clemens[1], So Young Chang[1], Xiaoxiao Jia[1], Hayley A. McQuilten[1], Ashley Huey Yiing Yap[1], Aira F. Cabug[1], Mitra Ashayeripanah[1], Hamish E. G. McWilliam[1], Jose A. Villadangos[1,3], Maximilien Evrard[1], Laura K. Mackay[1], Linda M. Wakim[1], John K. Fazakerley[1,2*], Katherine Kedzierska[1#*] and Lukasz Kedzierski[1#*]

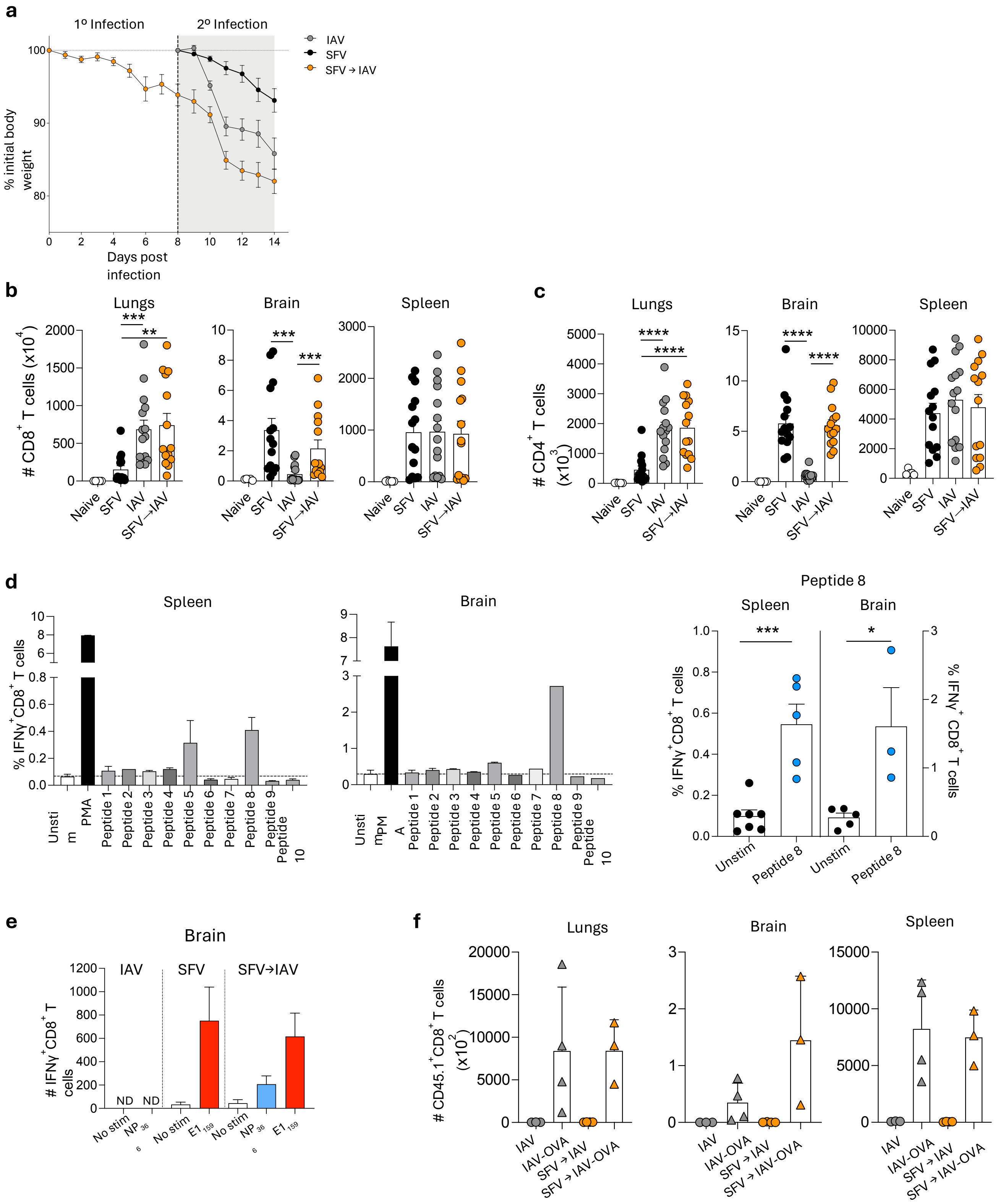
a
1º Infection
2º Infection
IAV
SFV
SFV → IAV
% initial body weight
Days post infection
b
Lungs
Brain
Spleen
CD8+ T cells (x10^4)
Naive
SFV
IAV
SFV→IAV
c
CD4+ T cells (x10^3)
d
% IFNγ+CD8+ T cells
Unstim
PMA
Peptide 1
Peptide 2
Peptide 3
Peptide 4
Peptide 5
Peptide 6
Peptide 7
Peptide 8
Peptide 9
Peptide 10
Peptide 8
e
IFNγ+CD8+ T cells
ND
No stim
NP366
E1159
f
CD45.1+CD8+ T cells (x10^2)
IAV-OVA
SFV → IAV
SFV → IAV-OVA

Supplementary Figure S1. Screening for immunogenic SFV epitopes. **a** Weight loss in SFV→IAV infected mice was monitored for 14 days, whereas IAV and SFV infected mice were monitored for 7 days (n= 14-15, error bars represent SEM). Absolute numbers of **b** $CD8^+$ T cells and **c** $CD4^+$ T cells across different anatomical sites in naïve, SFV, IAV, and SFVgIAV infected mice (n=14-15, error bar represents SEM). **d** Frequencies of IFN-$\gamma^+$$CD8^+$ T cells in the spleen and brain after SFV infection. Cells from spleen and brain were isolated 8 days post-infection and restimulated with individual peptides (Peptide 1-5, n = 2; Peptide 6-10, n = 3). Dotted line indicates level of cytokine production in DMSO background control. Error bars represent SEM. Right panel; pooled data from 2 independent experiments confirming immunodominance of Peptide 8 (K^b-$E1_{159\text{-}166}$). **e** Intracellular cytokine staining of $CD8^+$ T cells isolated on 7 dpi from the brain of IAV, SFV or SFVgIAV infected mice, and stimulated with a cognate peptide. Absolute numbers of IFNγ producing cells are plotted (n = 4-5, error bars represent SD). **f** Numbers of OT-I cells ($CD8^+CD45.1^+$) in the lung, brain and spleen following adoptive transfer of naïve OT-I cells. $1\text{x}10^6$ of OT-I cells were transferred into naïve mice or mice previously infected with SFV on d7 post-infection. One day after the transfer, the recipient mice were infected with $1\text{x}10^4$ pfu of either IAV-OVA or wild-type IAV. Organs were harvested on d7 after the IAV infection. Error bars represent SD. Significance was determined by unpaired Student's t-test, *<0.05, **<0.005, ***<0.001, ****<0.0001.

a

$D^{b}NP_{366}{}^{+}$ $CD8^{+}$ T cells

Lung

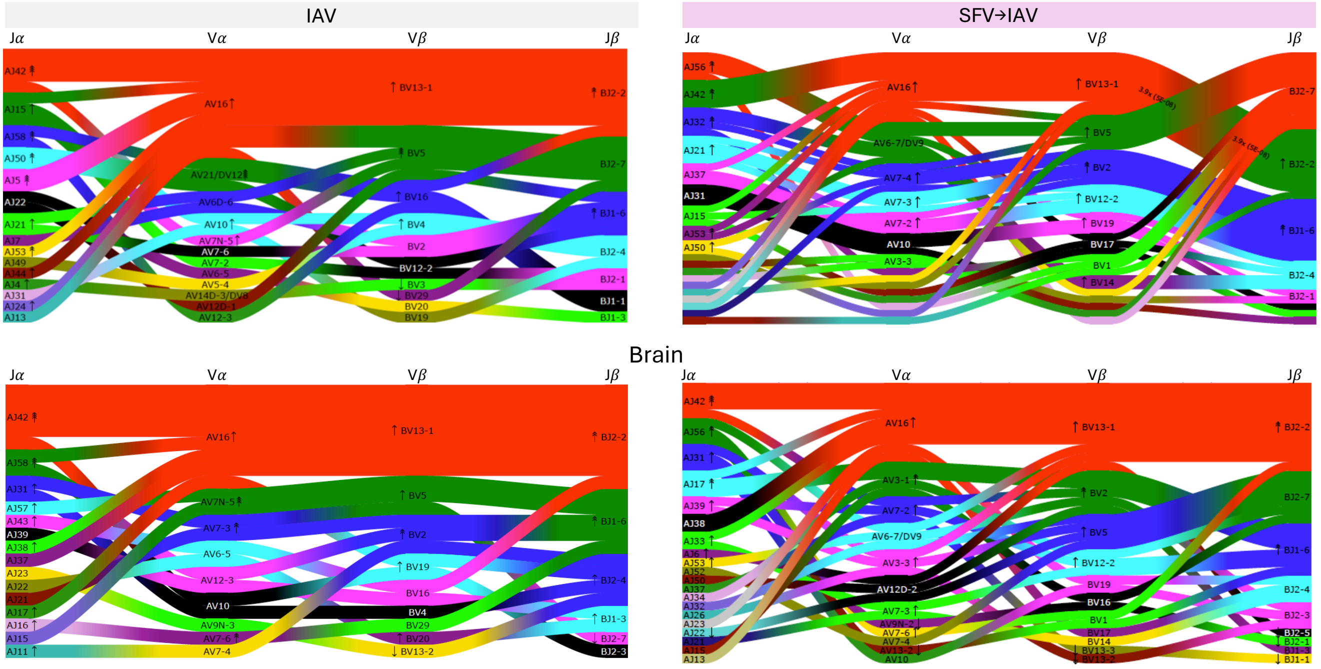

b

$D^{b}PA_{224}{}^{+}$ $CD8^{+}$ T cells

Lung

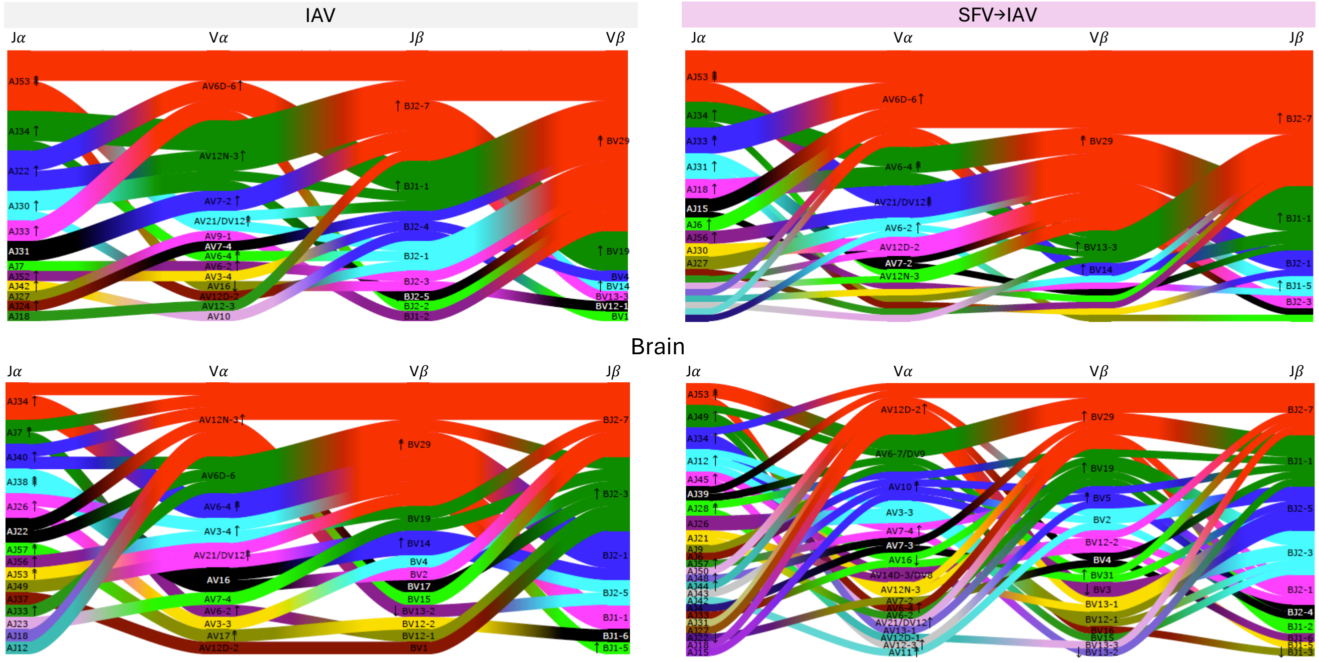

Supplementary Figure S2. V and J gene segment usage and covariation in IAV-specific responses within (a) $D^bNP_{366}{}^+CD8^+$ and (b) $D^bPA_{224}{}^+CD8^+$ T cells in the lung and brain of IAV and SFV→IAV infected mice. Gene segment usage is shown in vertical stacks, with gene-gene pairing landscapes shown by curved segments, with thickness being proportional to the TCR numbers with the gene pairing). Up or down arrows reflect the enrichment of gene segments relative to background, with each arrowhead indicating a 2-fold enrichment. The clonally expanded TCRs were reduced to a single data point for this analysis. Genes are coloured based on frequency: red (most frequent), green (second most frequent), blue, cyan, magenta, and black, followed by assorted colours for rare frequencies.

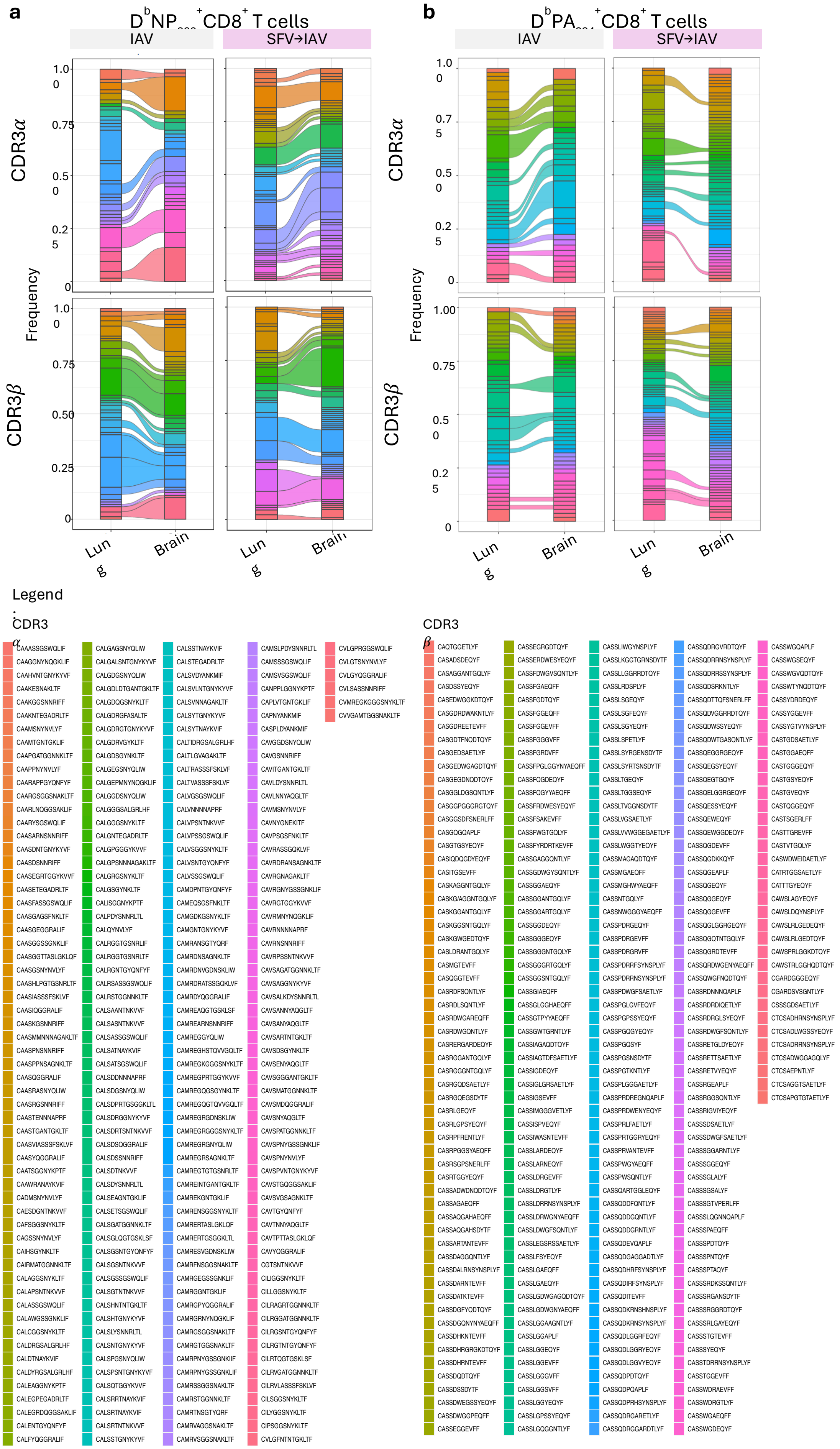
a
D^bNP366+CD8+ T cells
IAV
SFV→IAV
CDR3α
CDR3β
Frequency
1.00
0.75
0.50
0.25
0
Lung
Brain
b
D^bPA224+CD8+ T cells
IAV
SFV→IAV
CDR3α
CDR3β
Frequency
1.00
0.75
0.50
0.25
0
Lung
Brain
Legend:
CDR3α
CDR3β

Supplementary Figure S3. Alluvial plots showing the frequency of CDR3α/β clonotypes in IAV-specific responses within (a) $D^bNP_{366}{}^+$ $CD8^+$ and (b) $D^bPA_{224}{}^+$ $CD8^+$ T cells from the lung and brain of IAV and SFV→IAV infected mice. The connections between the bars represent CDR3 clonotypes shared between lungs and brains in each infection type.

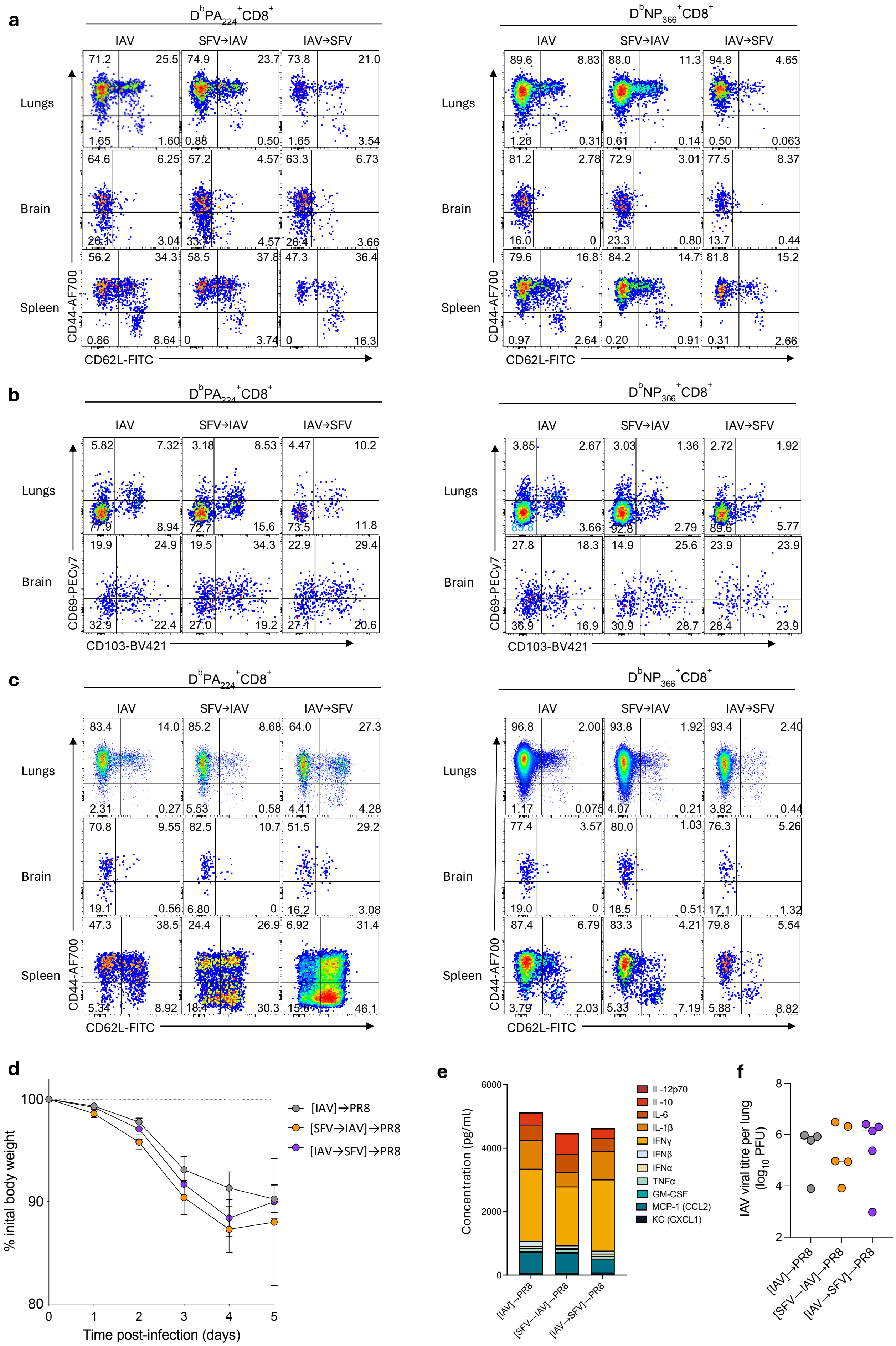

a
DbPA224+CD8+
IAV
SFV→IAV
IAV→SFV
Lungs
71.2 25.5 1.65 1.60
74.9 23.7 0.88 0.50
73.8 21.0 1.65 3.54
Brain
64.6 6.25 26.1 3.04
57.2 4.57 33.4 4.57
63.3 6.73 26.4 3.66
Spleen
56.2 34.3 0.86 8.64
58.5 37.8 0 3.74
47.3 36.4 0 16.3
CD44-AF700
CD62L-FITC
DbNP366+CD8+
IAV
SFV→IAV
IAV→SFV
Lungs
89.6 8.83 1.26 0.31
88.0 11.3 0.61 0.14
94.8 4.65 0.50 0.063
Brain
81.2 2.78 16.0 0
72.9 3.01 23.3 0.80
77.5 8.37 13.7 0.44
Spleen
79.6 16.8 0.97 2.64
84.2 14.7 0.20 0.91
81.8 15.2 0.31 2.66
CD44-AF700
CD62L-FITC
b
DbPA224+CD8+
IAV
SFV→IAV
IAV→SFV
Lungs
5.82 7.32 77.9 8.94
3.18 8.53 72.7 15.6
4.47 10.2 73.5 11.8
Brain
19.9 24.9 32.9 22.4
19.5 34.3 27.0 19.2
22.9 29.4 27.1 20.6
CD69-PECy7
CD103-BV421
DbNP366+CD8+
IAV
SFV→IAV
IAV→SFV
Lungs
3.85 2.67 89.8 3.66
3.03 1.36 92.8 2.79
2.72 1.92 89.6 5.77
Brain
27.8 18.3 36.9 16.9
14.9 25.6 30.9 28.7
23.9 23.9 28.4 23.9
CD69-PECy7
CD103-BV421
c
DbPA224+CD8+
IAV
SFV→IAV
IAV→SFV
Lungs
83.4 14.0 2.31 0.27
85.2 8.68 5.53 0.58
64.0 27.3 4.41 4.28
Brain
70.8 9.55 19.1 0.56
82.5 10.7 6.80 0
51.5 29.2 16.2 3.08
Spleen
47.3 38.5 5.34 8.92
24.4 26.9 18.4 30.3
6.92 31.4 15.6 46.1
CD44-AF700
CD62L-FITC
DbNP366+CD8+
IAV
SFV→IAV
IAV→SFV
Lungs
96.8 2.00 1.17 0.075
93.8 1.92 4.07 0.21
93.4 2.40 3.82 0.44
Brain
77.4 3.57 19.0 0
80.0 1.03 18.5 0.51
76.3 5.26 17.1 1.32
Spleen
87.4 6.79 3.79 2.03
83.3 4.21 5.33 7.19
79.8 5.54 5.88 8.82
CD44-AF700
CD62L-FITC
d
% inital body weight
100
90
80
0 1 2 3 4 5
Time post-infection (days)
[IAV]→PR8
[SFV→IAV]→PR8
[IAV→SFV]→PR8
e
Concentration (pg/ml)
6000
4000
2000
0
[IAV]→PR8
[SFV→IAV]→PR8
[IAV→SFV]→PR8
IL-12p70
IL-10
IL-6
IL-1β
IFNγ
IFNβ
IFNα
TNFα
GM-CSF
MCP-1 (CCL2)
KC (CXCL1)
f
IAV viral titre per lung (log10 PFU)
8
6
4
2
[IAV]→PR8
[SFV→IAV]→PR8
[IAV→SFV]→PR8

Supplementary Figure S4. Cytokine profile and viral load following H1N1 PR8 re-challenge. All primed mice were subjected to i.n. infection with 10^3 pfu H1N1 PR8 100 days post-co-infection. At d5 following IAV-PR8 challenge, lung, brain and spleen tissues were harvested from all experimental groups. **a** Concatenated FACS plots (n=5) of IAV-specific T_{EM} ($CD62L^{lo}CD44^{hi}$) and T_{CM} ($CD62L^{hi}CD44^{hi}$) of $D^bPA_{224}{}^+CD8^+$ T cell (left) and $D^bNP_{366}{}^+CD8^+$ T cell (right) specificities across different anatomical sites of IAV, SFV→IAV, and IAV→SFV infected mice. **b** Concatenated FACS plots (n=5) of IAV-specific T_{RM} ($CD103^+CD69^+$) of $D^bPA_{224}{}^+CD8^+$ T cell (left) and $D^bNP_{366}{}^+CD8^+$ T cell (right) specificities in the lungs and brain of IAV, SFV→IAV, and IAV→SFV infected mice. **c** Concatenated FACS plots (n=5) of IAV-specific $CD8^+$ effector T cells ($CD62L^{lo}CD44^{hi}$) for $D^bNP_{366}{}^+CD8^+$ and $D^bPA_{224}{}^+CD8^+$ T cells across different anatomical sites of IAV, SFV→IAV, and IAV→SFV PR8 challenged mice. **d** Weight loss of [IAV]→PR8, [SFV→IAV]→PR8, and [IAV→SFV]→PR8 challenged mice were monitored for 5 days (n= 9-10, error bars represent SEM). **e** Cytokine levels in the lungs were measured by Legendplex, and **f** lung viral loads were determined by plaque assay.

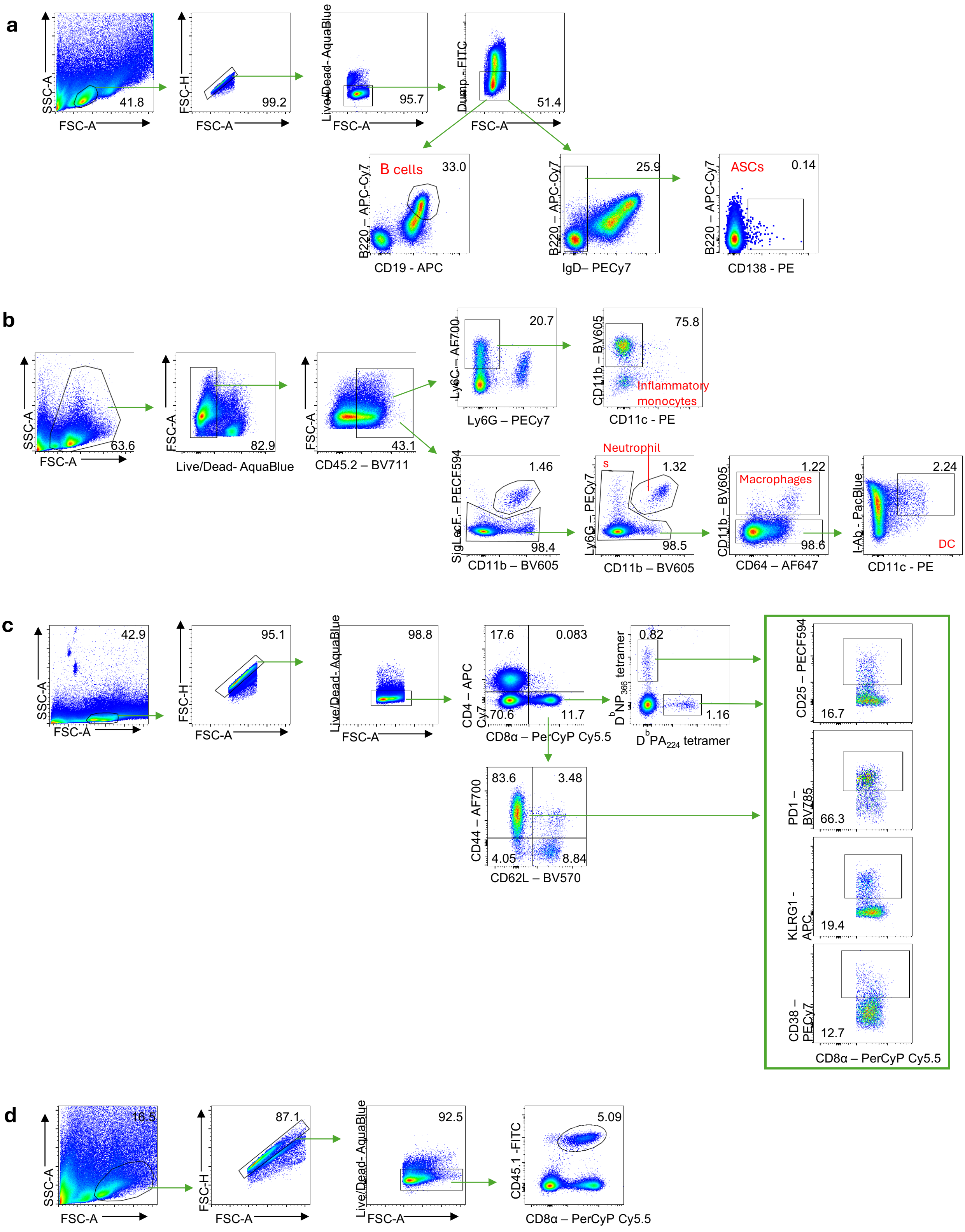
a
SSC-A
FSC-A
41.8
FSC-H
99.2
Live/Dead- AquaBlue
95.7
Dump - FITC
51.4
B220 – APC-Cy7
B cells
33.0
CD19 - APC
25.9
IgD– PECy7
ASCs
0.14
CD138 - PE
b
63.6
82.9
Live/Dead- AquaBlue
43.1
CD45.2 – BV711
Ly6C – AF700
20.7
Ly6G – PECy7
CD11b – BV605
75.8
Inflammatory monocytes
CD11c - PE
SigLecF – PECF594
1.46
98.4
CD11b – BV605
Neutrophils
Ly6G – PECy7
1.32
98.5
Macrophages
1.22
98.6
CD64 – AF647
I-Ab - PacBlue
2.24
DC
CD11c - PE
c
42.9
95.1
98.8
CD4 – APC Cy7
17.6
0.083
70.6
11.7
CD8α – PerCyP Cy5.5
0.82
1.16
CD44 – AF700
83.6
3.48
4.05
8.84
CD62L – BV570
CD25 – PECF594
16.7
PD1 – BV785
66.3
KLRG1 - APC
19.4
CD38 – PECy7
12.7
d
16.5
87.1
92.5
CD45.1 -FITC
5.09

Supplementary Figure S5. Gating strategy for flow cytometric analysis of brain infiltrates, lungs, and spleen and flow cytometric analysis of OT-I cells. Combination of antibodies against surface markers (see Methods section) were used in 3 panels to gate on **a** B cells, **b** myeloid cells and **c** T cells. Values shown indicate the percentage of gated or positive cells. **d** $1x10^6$ of *in vitro* activated or naïve OT-I cells were transferred into recipient mice. One day after the transfer, the recipient mice were infected with $1x10^4$ pfu of either IAV-OVA or wild-type IAV. Brains, lungs and spleens were harvested on d7 after the IAV infection.

Supplementary Table 1. Frequency of public and recurrent CDR3αβ amino acid clonotypes within (A) $D^bNP_{366}{}^+CD8^+$ and (B) $D^bPA_{224}{}^+CD8^+$ T cells in the lung and brain of IAV and SFV→IAV infected mice.

							IAV								SFV→IAV									
							M1		M2		M3		M4		M1		M2		M3		M4		M5	
	CDR3β	TRBV	TRBJ	CDR3α	TRAV	TRAJ	B	L	B	L	B	L	B	L	B	L	B	L	B	L	B	L	B	L
A	CASSGGSNTGQLYF	13	2-2	CAMRGSGGSKLTF	16/DV11	42			3	2			1						2		6	3		
	CASKGGANTGQLYF	13	2-2	CAMRESVGDNSKLIW	16/DV11	38									1	1	1							
	CASSQDLGGVYEQYF	5	2-7	CALRSTGGNNKLTF	6/DV9	56													7	7				
	CASSSRDKSSQNTLYF	17	2-4	CAMRGRNYNQGKLIF	16/DV11	23											6	6						
	CASRDWGQNTLYF	19	2-4	CAASDSNNRIFF	10	31			9	2														
	CGARDSVSGNTLYF	20	1-3	CVLGPRGGSWQLIF	6	22			8	2														
	CASSPDRRFSYNSPLYF	5	1-6	CAVSMDQGGRALIF	7	15	4	1																
	CASSPWSQNTLYF	4	2-4	CAAKESKLTF	10	42							1	3										
	CASRGGANTGQLYF	13	2-2	CAMREGQGTQVVGQLTF	16/DV11	5		3		1														
	CASSGGANTGQLYF	13	2-2	CAMRVSGGSKLTF	16/DV11	42	2	1																
	CTCSADWGGAGQLYF	1	2-2	CAIRMATGGNNKLTF	13/DV7	56									1	2								
	CASSLDRRNSYNSPLYF	12	1-6	CAAMTGNTGKLIF	7	37																	1	2
	CASSFSAKEVFF	16	1-1	CILRVLASSSFSKLVF	21/DV12	50		2		1														
	CASSQGQTNTGQLYF	16	2-2	CALSGLQGTGSKLSF	12	58							1	1										
	CASKAGGNTGQLYF	13	2-2	CANPPLGGNYKPTF	12	6									1	1								
	CASSSRGANSDYTF	17	1-2	CAVYQGGRALIF	3	15															1	1		
	CASSLDRRNSYNSPLYF	12	1-6	CAVMSNYNVLYF	7	21																	1	1
B	CATTTGYEQYF	13	2-7	CALDRGSALGRLHF	6	18												5		1				
	CASSWGDEQYF	29	2-7	CALSDPRTGSGGKLTL	12	44															1	2		
	CASSFYRDRTKEVFF	14	1-1	CALSDSQGGRALIF	6	15																2		1
	CASWDWEIDAETLYF	12	2-3	CAMSLPDYSNNRLTL	6	7	1	1																
	CASTGVEQYF	29	2-7	CALSRTNTNKVVF	12	34					1	1												
	CASSDWEGSSYEQYF	13	2-7	CALRGGTGSNRLTF	12	28									1	1								
	CASSLGGEVFF	29	1-1	CALGGGSNYKLTF	6/DV9	53																	1	1
	Number of sequences						14	31	34	23	18	13	7	13	25	13	19	26	37	24	17	37	11	26

M: mouse; L: lung; B: brain

Supplementary Table 2. Frequency of CDR3αβ clonotypes within $D^bNP_{366}{}^+CD8^+$ T cells detected in the lung and brain of IAV and SFV→IAV infected mice.

						IAV								SFV→IAV									
						M1		M2		M3		M4		M1		M2		M3		M4		M5	
CDR3β	TRBV	TRBJ	CDR3α	TRAV	TRAJ	B	L	B	L	B	L	B	L	B	L	B	L	B	L	B	L	B	L
**CASSPDRRFSYNSPLYF**	5	1-6	CAVSMDQGGRALIF	7	15	33.3%	5.6%																
**CASSQDLGGVYEQYF**	5	2-7	CAVSTGQGGSAKLIF	7	57	33.3%																	
**CASSGGANTGQLYF**	13	2-2	CAMRVSGGSNAKLTF	16/DV11	42	16.7%	5.6%																
**CASSGGGNTGQLYF**	13	2-2	CAMRVAGGSNAKLTF	16/DV11	42	8.3%																	
**CASGDRDWAKNTLYF**	13	2-4	CAVRMNYNQGKLIF	9	23	8.3%																	
**CASRDWGQNTLYF**	19	2-4	CAASDSNNRIFF	10	31			40.9%	12.5%														
**CGARDSVSGNTLYF**	20	1-3	CVLGPRGGSWQLIF	6	22			36.4%	6.3%														
**CASSGGSNTGQLYF**	13	2-2	CAMRGSGGSNAKLTF	16/DV11	42			13.6%	12.5%			20.0%						11.1%		54.5%	15.0%		
**CASKGGANTGQLYF**	13	2-2	CAMREGRGDNSKLIW	16/DV11	38			4.5%															
**CASSQDRRNSYNSPLYF**	2	1-6	CAGSSNYNVLYF	7	21			4.5%															
**CASKGGSNTGQLYF**	13	2-2	CAMREGRSAGNKLTF	16/DV11	17					25.0%													
**CASSIMGGGVETLYF**	19	2-3	CAMREKGNTGKLIF	16/DV11	37					25.0%													
**CASSQDSRKNTLYF**	2	2-4	CALSVNNAGAKLTF	12	39					12.5%													
**CASRGGGNTGQLYF**	13	2-2	CAMREAQGTGSKLSF	16/DV11	58					12.5%													
**CASSQDRRNSYNSPLYF**	2	1-6	CAVSDSGYNKLTF	7	11					12.5%													
**CASSLIWGYNSPLYF**	29	1-6	CAVRASSGQKLVF	7	16					12.5%													
**CASSPWSQNTLYF**	4	2-4	CAAKESNAKLTF	10	42							20.0%	60.0%										
**CASSQGQTNTGQLYF**	16	2-2	CALSGLQGTGSKLSF	12	58							20.0%	20.0%										
**CASSGWTGRNTLYF**	16	1-3	CALSDDNNNAPRF	6	43							20.0%											
**CASSQDIRFSYNSPLYF**	5	1-6	CAVRNSNNRIFF	7	31							20.0%											
**CASKG/AGGNTGQLYF**	13	12-2	CANPPLGGNYKPTF	12	6									12.5%									
**CASKAGGNTGQLYF**	13	2-2	CANPPLGGNYKPTF	12	6									12.5%	16.7%								
**CTCSADWGGAGQLYF**	1	2-2	CAIRMATGGNNKLTF	13/DV7	56									12.5%	33.3%								
**CASKGGANTGQLYF**	13	2-2	CAMRESVGDNSKLIW	16/DV11	38									12.5%	16.7%	6.3%							
**CASGEDSAETLYF**	13	2-3	CAVSANYAQGLTF	3	26									12.5%									
**CASSQDRRNSYNSPLYF**	2	1-6	CGTSNTNKVVF	3	34									12.5%									
**CASSQEGGRGEQYF**	5	2-7	CAASTGANTGKLTF	7	52									12.5%									

**CTCSADHRNSYNSPLYF**	1	1-6	CAVSPATGGNNKLTF	7	56		12.5%								
**CASSSRDKSSQNTLYF**	17	2-4	CAMRGRNYNQGKLIF	16/DV11	23			37.5%	50.0%						
**CASSDHRGRGKDTQYF**	13	2-5	CAVGSNNRIFF	3	31			12.5%							
**CASRDLSQNTLYF**	19	2-4	CAASDSNNRIFF	10	31			6.3%							
**CASSQEGSYEQYF**	5	2-7	CALRSASSGSWQLIF	12	22			6.3%							
**CASSQDLGGRYEQYF**	5	2-7	CAVRGNAGAKLTF	3	39			6.3%							
**CASSSRGGRDTQYF**	17	2-5	CALGNTEGADRLTF	6	45			6.3%							
**CASSDATKTEVFF**	13	1-1	CAARGSGGSNAKLTF	7	42			6.3%							
**CTCSADRRNSYNSPLYF**	1	1-6	CAAPGATGGNNKLTF	7	56			6.3%							
**CASSQDKRNSHNSPLYF**	2	1-6	CAVLDYSNNRLTL	7	7			6.3%							
**CASSQDLGGVYEQYF**	5	2-7	CALRSTGGNNKLTF	6/DV9	56					38.9%	46.7%				
**CASSQDRGARETLYF**	2	2-3	CALTRASSSFSKLVF	12	50					5.6%					
**CASSGGGRTGQLYF**	13	2-2	CAMRDNSAGNKLTF	16/DV11	17					5.6%					
**CASSIGLGRSAETLYF**	19	2-3	CAMREGGYQLIW	16/DV11	33					5.6%					
**CASSPWGYAEQFF**	16	2-1	CAVSVGSAGNKLTF	3	17					5.6%					
**CASSPDRRNSYNSPLYF**	2	1-6	CAVSVGSAGNKLTF	3	17					5.6%					
**CAWSTRLGGHQDTQYF**	31	2-5	CALVSNTGYQNFYF	6/DV9	49					5.6%					
**CASSQDLGGVYEQYF**	5	2-7	CAMRSTGGNNKLTF	6/DV9	56					5.6%					
**CASSQDRRNSYNSPLYF**	2	1-6	CAASMMNNNAGAKLTF	7	39					5.6%					
**CASSLARDEQYF**	16	2-7	CVLSASSNNRIFF	9	31					5.6%					
**CASSQDLGGVYEQYF**	5	2-7	CAMRGSGGSNAKLTF	16/DV11	42							9.1%			
**CASSSRGANSDYTF**	17	1-2	CAVYQGGRALIF	3	15							9.1%	5.0%		
**CASSQDDGRNTLYF**	2	1-3	CALGDQGSNYKLTF	6/DV9	53							9.1%			
**CASSGGSNTGQLYF**	13	2-2	CALAWGSSGNKLIF	6	32							9.1%			
**CASSSGGARNTLYF**	14	2-4	CAVGGDSNYQLIW	7	33							9.1%			
**CASSGGANTGQLYF**	13	2-2	CAMRTNSGTYQRF	16/DV11	13									28.6%	
**CASSGGSNTGQLYF**	13	2-2	CAMRGTGGSNAKLTF	16/DV11	42									28.6%	
**CASSPRVANTEVFF**	13	1-1	CAMEQSGSFNKLTF	13	4									14.3%	
**CASSLDRRNSYNSPLYF**	12	1-6	CAVMSNYNVLYF	7	21									14.3%	7.7%
CASSLDRRNSYNSPLYF	12	1-6	CAAMTGNTGKLIF	7	37									14.3%	15.4%
CASSQDLGGVYEQYF	5	2-7	CAMREGKGGGSNYKLTF	16/DV11	53	44.4%									

**CASRGGANTGQLYF**	13	2-2	CAMREGQGTQVVGQLTF	16/DV11	5	16.7%	6.3%						
**CASSFSAKEVFF**	16	1-1	CILRVLASSSFSKLVF	21/DV12	50	11.1%	6.3%						
**CASRLGPSYEQYF**	29	2-7	CAMRERTGSGGKLTL	16/DV11	44	5.6%							
**CASSQDLGGVYEQYF**	5	2-7	CVMREGKGGGSNYKLTF	16/DV11	53	5.6%							
**CASSPGTKNTLYF**	4	2-4	CAARAPPGYQNFYF	5	49	5.6%							
**CASSPDRRNSYNSPLYF**	12	1-6	CAASQGGRALIF	7	15		12.5%						
**CASSPRTGGRYEQYF**	5	2-7	CALASSGSWQLIF	12	22		6.3%						
**CGARDGGGEQYF**	20	2-7	CAVRNNNNAPRF	13	43		6.3%						
**CASSLDRWGNYAEQFF**	3	2-1	CAASGAGSFNKLTF	14/DV8	4		6.3%						
**CASAGGANTGQLYF**	13	2-2	CAMRANSGTYQRF	16/DV11	13		6.3%						
**CASSQELGGRGEQYF**	5	2-7	CAMRERTASLGKLQF	16/DV11	24		6.3%						
**CASSQDRRNSYNSPLYF**	2	1-6	CALQYNVLYF	6	21		6.3%						
**CGARDSVSGNTLYF**	20	1-2	CVLGPRGGSWQLIF	6	22		6.3%						
**CASSFPGLGGYNYAEQFF**	12	2-1	CILRTQGTGSKLSF	21/DV12	58			37.5%					
**CASSQARTGGLEQYF**	5	2-7	CALSLYSNNRLTL	6	7			25.0%					
**CASSGGGNTGQLYF**	13	2-2	CAMRDYQGGRALIF	16/DV11	15			12.5%					
**CASKGGANTGQLYF**	13	2-2	CAMRGEGSSGNKLIF	16/DV11	32			12.5%					
**CASSGGGNTGQLYF**	13	2-2	CAMRSSGGSNAKLTF	16/DV11	42			12.5%					
**CASSQDRRNSYNSPLYF**	2	1-6	CAVSPNYNVLYF	7	21				20.0%				
**CASRDWGAREQFF**	19	2-1	CAASPNSNNRIFF	10	31					16.7%			
**CASSQDRRNSYNSPLYF**	2	1-6	CAASFASSGSWQLIF	7	22					16.7%			
**CASRDFSQNTLYF**	19	2-4	CAASDSNNRIFF	10	31						33.3%		
**CASRDFSQNTLYF**	19	2-4	CAASDSNNRIFF	10	31						8.3%		
**CASSTDRRNSYNSPLYF**	12	1-6	CAAMSNYNVLYF	7	21						8.3%		
**CASRGGGNTGQLYF**	13	2-2	CAVRPSSNTNKVVF	7	34							20.0%	
**CASRGGANTGQLYF**	13	2-2	CAMRGGNTGKLIF	16/DV11	37							6.7%	
**CTCSADLWGSSYEQYF**	1	2-7	CAVRDRANSAGNKLTF	1	17							6.7%	
**CASRLGEQYF**	13	2-7	CAVSGGGANTGKLTF	3	52							6.7%	
**CASSLGGYEQYF**	29	2-7	CALRSTGGNNKLTF	6/DV9	56							6.7%	
**CTCSADLWGSSYEQYF**	1	2-7	CAVRGNYGSSGNKLIF	7	32							6.7%	
**CASSSGGARNTLYF**	14	2-4	CALAWGSSGNKLIF	6/DV9	32								30.0%

**CASSRIGVIYEQYF**	17	2-7	CAASIASSSFSKLVF	14/DV8	50																15.0%		
**CASSQDLGGVYEQYF**	5	2-7	CAMREGRGGGSNYKLTF	16/DV11	53																10.0%		
**CASSPLGGGAETLYF**	19	2-3	CALGDQGSNYKLTF	6/DV9	53																10.0%		
**CASSDHKNTEVFF**	13	1-1	CAVSPVNTGNYKYVF	3	40																5.0%		
**CASSPLGGGAETLYF**	19	2-3	CAMGDKGSNYKLTF	6/DV9	53																5.0%		
**CASSQDRRNSYNSPLYF**	2	1-6	CADMSNYNVLYF	7	21																5.0%		
**CASRGGGNTGQLYF**	13	2-2	CAMRFNSGGSNAKLTF	16/DV11	42																		15.4%
CASSDWGGPEQFF	16	2-1	CAAKGGSNNRIFF	10	31																		7.7%
CASSGGANTGQLYF	13	2-2	CAMRGPYQGGRALIF	16/DV11	15																		7.7%
CASRGGANTGQLYF	13	2-2	CAMREGRGNYQLIW	16/DV11	33																		7.7%
CASSPGPSSYEQYF	12	2-7	CAMRGTGGSNAKLTF	16/DV11	42																		7.7%
CASSQGLGGRGEQYF	5	2-7	CALVNNNNAPRF	6	43																		7.7%
CASSQDRRNSYNSPLYF	2	1-6	CAASYQGGRALIF	7	15																		7.7%
CASSQDRRNSYNSPLYF	2	1-6	CAAPPNYNVLYF	7	21																		7.7%
CASSQDRRNSYNSPLYF	2	1-6	CAASVIASSSFSKLVF	7	50																		7.7%
**Total proportion of sequences**						100.0 %	100.0 %	100.0 %	100.0 %	100.0 %	100.0 %	100.0 %	100.0 %	100.0 %	100.0 %	100.0 %	100.0 %	100.0 %	100.0 %	100.0 %	100.0 %	100.0 %	100.0 %

M: mouse; L: lung; B: brain

Supplementary Table 3. Frequency of CDR3αβ clonotypes within $D^bPA_{224}{}^+CD8^+$ T cells detected in the lung and brain of IAV and SFV→IAV infected mice.

						**IAV**								**SFV→IAV**									
						M1		M2		M3		M4		M1		M2		M3		M4		M5	
CDR3β	TRB V	TRB J	CDR3α	TRAV	TRA J	B	L	B	L	B	L	B	L	B	L	B	L	B	L	B	L	B	L
CASWDWEIDAETLYF	12	2-3	CAMSLPDYSNNRLTL	6	7	50.0%	7.7%																
CASSIAGTDFSAETLYF	19	2-3	CAAGGNYNQGKLIF	7	23	50.0%																	
CASSWGQAPLF	29	1-5	CALVPSSGSWQLIF	6/DV9	22			16.7%															
CASSSPAEQFF	29	2-1	CALSQTGGYKVVF	12	12			8.3%															
CASSRDRDIQETLYF	17	2-3	CALDRGSALGRLHF	12	18			8.3%															
CASSPRDWENYEQYF	15	2-7	CALSGSSGSWQLIF	12	22			8.3%															
CTCSAGGTSAETLYF	1	2-3	CALSEAGNTGKLIF	12	37			8.3%															
CASSADWDNQDTQYF	14	2-5	CAMRDNVGDNSKLIW	16/DV11	38			8.3%															
CASSQEWGGDEQYF	2	2-7	CAMRDNVGDNSKLIW	16/DV11	38			8.3%															
CASSLRDSPLYF	12	1-6	CALEGRDQGGSAKLIF	17	57			8.3%															
CASTGDSAETLYF	4	2-3	CAVTNNYAQGLTF	3	26			8.3%															
CASSPDRGRVFF	29	1-1	CAVSAGATGGNNKLTF	3	56			8.3%															
CAQTGGETLYF	29	2-3	CALGAGSNYQLIW	6/DV9	33			8.3%															
CASSLSGFEQYF	29	2-7	CALVPSNTNKVVF	6	34					30.0%													
CASTGVEQYF	29	2-7	CALSRTNTNKVVF	12	34					10.0%	20.0%												
CASSGGAEQYF	29	2-7	CALSNTGNYKYVF	12	40					10.0%													
CASGEGDNQDTQYF	13	2-5	CALPDYSNNRLTL	12	7					10.0%													
CASSNWGGGYAEQFF	14	2-1	CILRGTNTGYQNFYF	21/DV12	49					10.0%													
CASSLGAEQFF	29	2-1	CILIGGSNYKLTF	21/DV12	53					10.0%													
CASRTGGYEQYF	19	2-7	CAVSNYAQGLTF	3	26					10.0%													
CASSGIAEQFF	29	2-1	CALVPSNTNKVVF	6	34					10.0%													
CASSLDRGEVFF	29	1-1	CALGDRGTGNYKYVF	6/DV9	40							50.0%											
CASSFGAEQFF	29	2-1	CALGDRGTGNYKYVF	6/DV9	40							50.0%											
CASSRDNNNQAPLF	12	1-5	CAASTENNNAPRF	10	43									11.8%									
CASSQDVGGRRDTQYF	2	2-5	CAASHLPGTGSNRLTF	10	28									5.9%									
CASSQDGAGGADTLYF	2	2-4	CVVGAMTGGSNAKLTF	11	42									5.9%									
CASSYDRDEQYF	29	2-7	CALSHNTNTGKLTF	12	27									5.9%									

CASSDWEGSSYEQYF	13	2-7	CALRGGTGSNRLTF	12	28	5.9%	14.3%			
CASSAGAEQFF	19	2-1	CALSASNTNKVVF	12	34	5.9%				
CASSEGRGDTQYF	13	2-5	CALTLGVAGAKLTF	12	39	5.9%				
CASSQDRGGARDTLYF	5	2-4	CALSTEGADRLTF	12	45	5.9%				
CASSDSSDYTF	29	1-2	CALSGSNTGYQNFYF	12	49	5.9%				
CASSLWGGTYEQYF	12	2-7	CAASETEGADRLTF	14/DV8	45	5.9%				
CASSQRDWGENYAEQFF	12	2-1	CAMREGPRTGGYKVVF	16/DV11	12	5.9%				
CASSLLGGRRDTQYF	12	2-5	CAVRGTGGYKVVF	3	12	5.9%				
CAWSLRLGEDTQYF	31	2-5	CALRGNTGYQNFYF	6/DV9	49	5.9%				
CASSLGQGGNTLYF	16	1-3	CALGDRVGYKLTF	6/DV9	9	5.9%				
CASTGGGEQYF	29	2-7	CALGPSNNNAGAKLTF	6	39	5.9%				
CAWSLRLGEDEQYF	31	7-1	CAVPSGSFNKLTF	7	4	5.9%				
CASSPDWGFSAETLYF	3	2-3	CAESDGNTNKVVF	12	34			33.3%		
CASSIWASNTEVFF	19	1-1	CALENTGYQNFYF	13	49			33.3%		
CASMGTEVFF	29	1-1	CALVPSNTNKVVF	6	34			33.3%		
CASSIGLGRSAETLYF	19	2-3	CAMREGGYQLIW	16/DV11	33				21.1%	
CASSDGFYQDTQYF	13	2-5	CAASGSNYNVLYF	14/DV8	21				10.5%	
CASRPGGSYAEQFF	5	2-1	CAASIQGGRALIF	10	15				5.3%	
CASRERGARDEQYF	13	2-7	CALSDRTSNTNKVVF	12	34				5.3%	
CASSQDRGVRDTQYF	2	2-5	CALTVASSSFSKLVF	12	50				5.3%	
CASSFDWGVSQNTLYF	3	2-4	CALSDYSNNRLTL	12	7				5.3%	
CASSIGLGRSAETLYF	19	2-3	CAMREGGYQLIW	16/DV11	33				5.3%	
CASSSDSAETLYF	12	2-3	CAVSENYAQGLTF	3	26				5.3%	
CASSYGGEVFF	29	1-1	CAFSGGSNYKLTF	6	53				5.3%	
CASGDREETEVFF	13	1-1	CAASEGRTGGYKVVF	7	12				5.3%	
CASSQGRDTEVFF	4	1-1	CAAASSGSWQLIF	7	22				5.3%	
CASSLFSYEQYF	29	2-7	CAVNYGNEKITF	7	48				5.3%	
CASSSRLGAYEQYF	29	2-7	CAVTGYQNFYF	7	49				5.3%	
CASSQDITEVFF	5	1-1	CAARLNQGGSAKLIF	7	57				5.3%	
CAWSLRLGEDTQYF	31	2-5	CALGSGYNKLTF	9	11				5.3%	
CASSYGTVYNSPLYF	4	1-6	CALTIDRGSALGRLHF	12	18					16.7%

CASSSDWGFSAETLYF	3	2-3	CALSDSSNNRIFF	12	31					16.7%			
CASSWGDEQYF	29	2-7	CALSDPRTGSGGKLTL	12	44					16.7%	11.8%		
CASSQDDFQNTLYF	2	2-4	CAPLVTGNTGKLIF	13/DV7	37					16.7%			
CASSFGGEQFF	29	2-1	CILIGGSNYKLTF	21/DV12	53					16.7%			
CASSPLGGGAETLYF	19	2-3	CVLGTSNYNVLYF	6	21					16.7%			
CASSPGSNSDYTF	19	1-2	CAATSGGNYKPTF	10	6							25.0%	
CASRGQDSAETLYF	15	2-3	CAVSANNYAQGLTF	3	26							25.0%	
CASSLGGEVFF	29	1-1	CALGGGSNYKLTF	6/DV9	53							25.0%	7.7%
CASITGSEVFF	29	1-1	CALGRGSNYKLTF	6/DV9	53							25.0%	
CASSLGGEQYF	29	2-7	CALSGTNTNKVVF	12	34	30.8%							
CASTGGAEQFF	29	2-1	CALSRTNAYKVIF	12	30	7.7%							
CASSLGGAPLF	29	1-5	CALSPGSNYQLIW	12	33	7.7%							
CASSNTGQLYF	29	2-2	CALVSSGSWQLIF	6/DV9	22	7.7%							
CASSLTGEQYF	29	2-7	CALGEGSNYQLIW	6/DV9	33	7.7%							
CASSPGQSYF	29	2-7	CALGGGSNYKLTF	6/DV9	53	7.7%							
CTCSAEPNTLYF	1	2-4	CAASGGTTASLGKLQF	7	24	7.7%							
CASSIGDEQYF	19	2-7	CAASKGSNNRIFF	7	31	7.7%							
CASSIGDEQYF	19	2-7	CAASRGSNNRIFF	7	31	7.7%							
CASSERDWESYEQYF	13	2-7	CALSETSGSWQLIF	12	22		14.3%						
CASSFRDWESYEQYF	14	2-7	CALSGSSGSWQLIF	12	22		14.3%						
CASSFGGEVFF	29	1-1	CALSSTNAYKVIF	12	30		14.3%						
CASSFGGGVFF	29	1-1	CILYGGSNYKLTF	21/DV12	53		14.3%						
CASSGGDEQYF	29	2-7	CALGGGSNYKLTF	6/DV9	53		14.3%						
CASSGTPYYAEQFF	29	2-1	CALVPSNTNKVVF	6	34		14.3%						
CASSLSYRGENSDYTF	29	1-2	CAVSARTNTGKLTF	9	27		14.3%						
CASSGGGEQYF	19	2-7	CALSAANTNKVVF	12	34			40.0%					
CASSSTGTEVFF	29	1-1	CALGAGSNYQLIW	6	33			20.0%					
CASTVTGQLYF	29	2-2	CALCGGSNYKLTF	6	53			20.0%					
CASSSPDTQYF	29	2-5	CALSATNAYKVIF	12	30				25.0%				
CASSPWSQNTLYF	4	2-4	CAAKESNAKLTF	10	42				12.5%				
CASSISPVEQYF	19	2-7	CALDRGSALGRLHF	12	18				12.5%				

CASSFGAEQFF	29	2-1	CILSGGSNYKLTF	21/DV12	53	12.5%					
CASSWDRGTLYF	29	2-4	CAVITGANTGKLTF	3	52	12.5%					
CASSQGGEVFF	29	1-1	CALVGSGSWQLIF	6	22	12.5%					
CASSLGGGVFF	29	1-1	CALGGGSNYKLTF	6	53	12.5%					
CASSDWEGSSYEQYF	13	2-7	CALRGGTGSNRLIF	12	28		14.3%				
CASDSSYEQYF	29	2-7	CALSHTGNYKYVF	12	40		14.3%				
CASSWGSEQYF	29	2-7	CALISGGNYKPTF	6/DV9	6		14.3%				
CASSLSGYEQYF	29	2-7	CALVPSNTNKVVF	6	34		14.3%				
CASSWGSEQYF	29	2-7	CALISGGNYKPTF	6	6		14.3%				
CASSIGSEVFF	19	1-1	CAASRGSNNRIFF	7	31		14.3%				
CATTTGYEQYF	13	2-7	CALDRGSALGRLHF	6	18			35.7%	11.1%		
CASADSDEQYF	29	2-7	CALSYTNAYKVIF	12	30			14.3%			
CASSLTGGSEQYF	16	2-7	CAMDPNTGYQNFYF	13	49			7.1%			
CASSSYEQYF	29	2-7	CALGEGSNYQLIW	6/DV9	33			7.1%			
CASSSGLALYF	29	2-3	CALGGGSNYKLTF	6/DV9	53			7.1%			
CASSSGSALYF	29	2-3	CALGGGSNYKLTF	6/DV9	53			7.1%			
CATTTGYEQYF	13	2-7	CALDRGSALGRLHF	6	18			7.1%			
CASSSYEQYF	29	2-7	CALGEGSNYQLIW	6	33			7.1%			
CASSWGSEQYF	29	2-7	CALGGGSNYKLTF	6	53			7.1%			
CASSWGAEQFF	29	2-1	CILSGGSNYKLTF	21/DV12	53				22.2%		
CASSWDRAEVFF	29	1-1	CILRVGATGGNNKLTF	21/DV12	56				22.2%		
CASSLSGEQYF	29	2-7	CALGEGSNYQLIW	6/DV9	33				11.1%		
CASSWGAEQFF	29	2-1	CALSGATGGNNKLTF	6/DV9	56				11.1%		
CASSLGGYEQYF	29	2-7	CALAPSNTNKVVF	6	34				11.1%		
CASSLGGYEQYF	29	2-7	CALVPSNTNKVVF	6	34				11.1%		
CASGQGQAPLF	29	1-5	CALGAGSNYQLIW	6/DV9	33					11.8%	
CASSFYRDRTKEVFF	14	1-1	CALSDSQGGRALIF	6	15					11.8%	7.7%
CASSQDEVQAPLF	2	1-5	CAVSPNYGSSGNKLIF	7	32					11.8%	
CASSDGQNYNYAEQFF	13	2-1	CAASARNSNNRIFF	14	31					5.9%	
CASSFGGEQFF	29	2-1	CILIGGSNYKLTF	21/DV12	53					5.9%	
CASSFGRDVFF	29	1-1	CILLGGSNYKLTF	21/DV12	53					5.9%	

CASSLDRGTLYF	29	2-4	CILRAGRTGGNNKLTF	21/DV12	56																5.9%		
CASQGGTEVFF	29	1-1	CALGAGSNYQLIW	6/DV9	33																5.9%		
CASSQGEAPLF	29	1-5	CALEAGGNYKPTF	6/DV9	6																5.9%		
CASSTGGEVFF	29	1-1	CAMRDRATSSGQKLVF	6	16																5.9%		
CASGGLDGSQNTLYF	29	2-4	CALASSGSWQLIF	6	22																5.9%		
CASSRETGLDYEQYF	17	2-7	CVLGYQGGRALIF	9	15																5.9%		
CASTGSYEQYF	13	2-7	CVLGFNTNTGKLTF	6	27																		23.1%
CASSEGGEVFF	29	1-1	CAASKGSNNRIFF	7	31																		15.4%
CASSSPAEQFF	29	2-1	CALSRTNAYKVIF	12	30																		7.7%
CASTQGGEQYF	29	2-7	CALSGTNTNKVVF	12	34																		7.7%
CASSPDRGEVFF	29	1-1	CILRGGATGGNNKLTF	21/DV12	56																		7.7%
CASSLDRGEVFF	29	1-1	CILRVGATGGNNKLTF	21/DV12	56																		7.7%
CASSSGGEQYF	29	2-7	CALGALSNTGNYKYVF	6/DV9	40																		7.7%
CASLDRANTGQLYF	12	2-2	CVLGFNTNTGKLTF	6	27																		7.7%
**Total number of sequences**						100.0 %	100.0 %	100.0 %	100.0 %	100.0 %	100.0 %	100.0 %	100.0 %	100.0 %	100.0 %	100.0 %	100.0 %	100.0 %	100.0 %	100.0 %	100.0 %	100.0 %	100.0 %

M: mouse; L: lung; B: brain
