# Supplementary material for: Prior infection with unrelated neurotropic virus exacerbates influenza disease and impairs lung T cell responses
Source: Nat Commun. 2024 Mar 23;15:2619. doi: 10.1038/s41467-024-46822-7 (PMC10960853; doi:10.1038/s41467-024-46822-7)
Supplement: Supplementary file 3 — Reporting summary [file 41467_2024_46822_MOESM3_ESM.pdf]

nature portfolio

Corresponding author(s): Katherine Kedzierska
Lukasz Kedzierski

Last updated by author(s): 29/01/2024

Reporting Summary

Nature Portfolio wishes to improve the reproducibility of the work that we publish. This form provides structure for consistency and transparency in reporting. For further information on Nature Portfolio policies, see our Editorial Policies and the Editorial Policy Checklist.

Statistics

For all statistical analyses, confirm that the following items are present in the figure legend, table legend, main text, or Methods section.

n/a	Confirmed	
☐	☒	The exact sample size (*n*) for each experimental group/condition, given as a discrete number and unit of measurement
☐	☒	A statement on whether measurements were taken from distinct samples or whether the same sample was measured repeatedly
☐	☒	The statistical test(s) used AND whether they are one- or two-sided<br>*Only common tests should be described solely by name; describe more complex techniques in the Methods section.*
☐	☒	A description of all covariates tested
☐	☒	A description of any assumptions or corrections, such as tests of normality and adjustment for multiple comparisons
☐	☒	A full description of the statistical parameters including central tendency (e.g. means) or other basic estimates (e.g. regression coefficient) AND variation (e.g. standard deviation) or associated estimates of uncertainty (e.g. confidence intervals)
☐	☒	For null hypothesis testing, the test statistic (e.g. *F*, *t*, *r*) with confidence intervals, effect sizes, degrees of freedom and *P* value noted<br>*Give P values as exact values whenever suitable.*
☒	☐	For Bayesian analysis, information on the choice of priors and Markov chain Monte Carlo settings
☒	☐	For hierarchical and complex designs, identification of the appropriate level for tests and full reporting of outcomes
☒	☐	Estimates of effect sizes (e.g. Cohen's *d*, Pearson's *r*), indicating how they were calculated

Our web collection on statistics for biologists contains articles on many of the points above.

Software and code

Policy information about availability of computer code

Data collection	BD FACS DIVA v8.0.1
Data analysis	FlowJo v10.9.0, Prism v9, RStudio 2022.07.2+576 "Spotted Wakerobin" Release, LEGENDplex™ Data Analysis website (https://legendplex.qognit.com/workflow), IMGT/V-QUEST program version: 3.6.1, http://tools.iedb.org/mhci/, http://www.cbs.dtu.dk/services/HLArestrictor/ for peptide prediction, infinityFlow R package as described in (Evrard et al., 2023; https://doi.org/10.1016/j.immuni.2023.06.005), OMIQ cloud platform (omiq.ai).

For manuscripts utilizing custom algorithms or software that are central to the research but not yet described in published literature, software must be made available to editors and reviewers. We strongly encourage code deposition in a community repository (e.g. GitHub). See the Nature Portfolio guidelines for submitting code & software for further information.

Data

Policy information about availability of data

All manuscripts must include a data availability statement. This statement should provide the following information, where applicable:
- Accession codes, unique identifiers, or web links for publicly available datasets
- A description of any restrictions on data availability
- For clinical datasets or third party data, please ensure that the statement adheres to our policy

All data generated or analysed during this study are included in this published article (and its supplementary information files). Source data are provided with this paper as Source Date file 'Source data file Foo et al.xlsx'. All relevant data are also available from the authors. All the raw data are available upon the request.

The TCR sequences that support the findings of this study are available on the Mendeley database https://data.mendeley.com/datasets/6xb4j8xtv5/1
Reserved DOI: 10.17632/6xb4j8xtv5.1

Research involving human participants, their data, or biological material

Policy information about studies with human participants or human data. See also policy information about sex, gender (identity/presentation), and sexual orientation and race, ethnicity and racism.

Reporting on sex and gender	N/A
Reporting on race, ethnicity, or other socially relevant groupings	N/A
Population characteristics	N/A
Recruitment	N/A
Ethics oversight	N/A

Note that full information on the approval of the study protocol must also be provided in the manuscript.

Field-specific reporting

Please select the one below that is the best fit for your research. If you are not sure, read the appropriate sections before making your selection.

[x] Life sciences   [ ] Behavioural & social sciences   [ ] Ecological, evolutionary & environmental sciences

For a reference copy of the document with all sections, see nature.com/documents/nr-reporting-summary-flat.pdf

Life sciences study design

All studies must disclose on these points even when the disclosure is negative.

Sample size	Sample size was >3 as per typical experimental design, and mouse availability. Typical group size was usually 5 (n=5) to ascertain enough data points for statistical analysis.
Data exclusions	No data were excluded with the exception of FACS samples with less than 10 gated events. These samples were plotted but not included in the statistical analysis due to insufficient number of gated events. All data points are provided in the source file.
Replication	Mouse experiments were performed at least twice and data were reproducible. All data points are provided in the source file.
Randomization	Mice were assigned into experimental groups to achieve equal distribution of age and sex across experimental groups.
Blinding	Experiments were not blinded.

Reporting for specific materials, systems and methods

We require information from authors about some types of materials, experimental systems and methods used in many studies. Here, indicate whether each material, system or method listed is relevant to your study. If you are not sure if a list item applies to your research, read the appropriate section before selecting a response.

Materials & experimental systems

n/a	Involved in the study	
[ ]	[x]	Antibodies
[ ]	[x]	Eukaryotic cell lines
[x]	[ ]	Palaeontology and archaeology
[ ]	[x]	Animals and other organisms
[x]	[ ]	Clinical data
[x]	[ ]	Dual use research of concern
[x]	[ ]	Plants

Methods

n/a	Involved in the study	
[x]	[ ]	ChIP-seq
[ ]	[x]	Flow cytometry
[x]	[ ]	MRI-based neuroimaging

Antibodies

Antibodies used	We used commercially-available antibodies as per Materials and Methods; see "Tetramer and immunophenotypic staining".

Lymphocytes were stained with combinations of commercial fluorochrome-conjugated antibodies (clone; catalogue number; dilution):
BD Biosciences: anti-CD8-PerCyP Cy5.5 (53-67; 551162; 1:200), anti-CD44-Alexa Flour 700 (1M7; 560567; 1:200), anti-CD4-APC Cy7 (GK1.5; 552051; 1:500), anti-TCR -BV711 (H57-597; 563135; 1:200), anti-CD25-PECF594 (PC61; 562694; 1:200), anti-CD45.1-FITC (A20; 561871; 1:800), anti-CD62L-FITC (MEL-14; 561917; 1:200), anti-CD45.1-PE (A20; 553776; 1:200), anti-V2TCR-FITC (B20.1; 553288; 1:100), anti-CD45R-APCCy7 (RA3-6B2, 552094; 1:200), anti-CD38-BV711 (90; 740697; 1:200), anti-CD138-PE (281-2; 553714; 1:400), anti-CD11c-FITC (HL3; 557400; 1:1000), anti-Gr1-FITC (RB6-8C5; 553126; 1:400), anti-CD64-AF647 (X54-5/7.1; 558539; 1:150), anti-Ly6C-AlexaFlour700 (AL-21; 561237; 1:400), anti-CD11b-BV605 (M1/70; 563015; 1:600), anti-CD45.2-BV711 (104; 563685; 1:400), anti-CD11c-PE (HL3; 553802; 1:400), anti-SigLecF-PECF594 (E50-2440; 562757; 1:1200), anti-Ly6G-PECy7 (1A8; 560601; 1:1200).

BioLegend: anti-CD62L-BV570 (MEL-14; 104433; 1:200), anti-CD279-BV785 (29F.1A12; 135225; 1:200), anti-CD38-PECy7 (90; 102718; 1:500), anti-CD8-BV510 (53-6.7; 100752; 1:200), anti-CD103-BV421 (2E7; 121422; 1:200), anti-CD69-PECy7 (H1.2F3; 104512; 1:200), anti-CD62L-PECy7 (MEL-14; 104418; 1:200), anti-GL7-PerCyPCy5.5 (GL7; 144610; 1:200), anti-CD19-APC (6D5; 11512; 1:400), anti-I-Ab-PacBlue (AF6-120.1, 116422; 1:400), anti-IgD-PECy7 (11-26c.2a; 405720; 1:200), anti-CD3-FITC (145-2C11; 100306; 1:400), anti-F4/80-FITC (RB6-8C5; 553126; 1:150).

Invitrogen eBiosciences (anti-KLRG1-FITC (2F1; 11-5893-82; 1:200). Cell viability was determined by staining with either Live/Dead-Aqua 525 (L34966A, ThermoFisher, 1:800) or Live/Dead Fixable Near-IR (L10119, ThermoFisher, 1:800).

Validation	Each antibody used had a validated technical data sheet as per manufacturer's website showing positive staining as opposed to the negative staining of isotype control. All antibodies were titrated in our laboratory prior to their use. Multiplex assay was tested with samples with known high responses from previous assays. Multiplex assay was also validated by testing single "beads" alone in parallel. FACS positive staining is shown for each antibody used as shown in the FACS plots in the main figures and supplementary figures.

Eukaryotic cell lines

Policy information about cell lines and Sex and Gender in Research

Cell line source(s)	Vero cells and Madin-Darby canine kidney (MDCK) cells were obtained from ATCC (#CCL-81 and #CCL-34 respectively).
Authentication	The cell lines were not authenticated.
Mycoplasma contamination	All cell lines tested mycoplasma negative.
Commonly misidentified lines (See ICLAC register)	None used.

Animals and other research organisms

Policy information about studies involving animals; ARRIVE guidelines recommended for reporting animal research, and Sex and Gender in Research

Laboratory animals	C57Bl/6 mice, OT-I/Ly5.1 mice, animals were 6-8 weeks old
Wild animals	No wild animals were used in this study
Reporting on sex	For experiments involving adoptive transfer of OT-I T cells, only females mice were used for both donor and recipients to prevent sex mismatch rejection.
Field-collected samples	No field collected samples were used in the study
Ethics oversight	Animal experiments followed the NHMRC Code of Practice for the Care and Use of Animals for Scientific Purposes guidelines and were approved by the University of Melbourne Animal Ethics Committee (AEC 1714184 and 21319).

Note that full information on the approval of the study protocol must also be provided in the manuscript.

Flow Cytometry

Plots

Confirm that:

- [x] The axis labels state the marker and fluorochrome used (e.g. CD4-FITC).
- [x] The axis scales are clearly visible. Include numbers along axes only for bottom left plot of group (a 'group' is an analysis of identical markers).
- [x] All plots are contour plots with outliers or pseudocolor plots.
- [x] A numerical value for number of cells or percentage (with statistics) is provided.

Methodology

Sample preparation	Samples were prepared as described in Materials and Methods; see "Tissue sampling and cell preparation".
Instrument	BD LSR Fortessa was used for acquisition of data, BD FACS Aria III was used for cell sorting
Software	BD FACS Diva v8.0.1, FlowJo v10.9.0.
Cell population abundance	Single cell sorting was performed, which was confirmed by the presence of single TCR chains.
Gating strategy	Gating strategy has been described in Supplementary Figure S5.

- [x] Tick this box to confirm that a figure exemplifying the gating strategy is provided in the Supplementary Information.
